# Questions and controversies: the role of necroptosis in liver disease

**DOI:** 10.1038/cddiscovery.2016.89

**Published:** 2016-12-05

**Authors:** Lily Dara, Zhang-Xu Liu, Neil Kaplowitz

**Affiliations:** 1 Research Center for Liver Disease, Keck School of Medicine, Department of Medicine, University of Southern California, Los Angeles, CA, USA; 2 Division of GI/Liver, Department of Medicine, Keck School of Medicine, University of Southern California, Los Angeles, CA, USA

## Abstract

Acute and chronic liver injury results in hepatocyte death and turnover. If injury becomes chronic, the continuous cell death and turnover leads to chronic inflammation, fibrosis and ultimately cirrhosis and hepatocellular carcinoma. Controlling liver cell death both in acute injury, to rescue the liver from acute liver failure, and in chronic injury, to curb secondary inflammation and fibrosis, is of paramount importance as a therapeutic strategy. Both apoptosis and necrosis occur in the liver, but the occurrence of necroptosis in the liver and its contribution to liver disease is controversial. Necroptosis is a form of regulated necrosis which occurs in certain cell types when caspases (+/−cIAPs) are inhibited through the RIPK1-RIPK3 activation of MLKL. The occurrence of necroptosis in the liver has recently been examined in multiple liver injury models with conflicting results. The aim of this review is to summarize the published data with an emphasis on the controversies and remaining questions in the field.

## Facts

- Hepatocytes do not express RIPK3 under basal conditions.
- Blocking apoptosis in hepatocytes protects from cell death (with no switch to necroptosis).
- Necrostatin-1 (RIPK1 kinase inhibitor) has been shown to protect against many models of acute liver injury such as acetaminophen toxicity.

## Open Questions

- What are the RIPK1 platform functions in hepatocytes *versus* RIPK1 kinase functions?
- Does RIPK1 participate independent of the necrosome in hepatocyte death pathways?
- Does necroptosis occur in non-parenchymal cells (NPCs)?
- Can RIPK3 be induced in hepatocytes in chronic liver disease, and if so does this sensitize to necroptosis?
- Does RIPK3-independent, but MLKL-dependent necrosis exist in hepatocytes in liver diseases?

The liver is a complicated vital organ responsible for metabolic processes, protein synthesis, and clearance of toxins and xenobiotics. The liver is the largest solid organ and has an unparalleled regenerative capacity, high vascular capacitance (receiving 30% of cardiac output) and is a site of immune-privilege.^1,2^ Hepatocytes are the main parenchymal cells making up 80% of liver mass, with the other epithelial cell type, the cholangiocytes lining the bile ducts, being the minority. The NPCs despite being high in number (40% of total cells) contribute to only 7% of liver volume because of their small size. These cells include the resident liver macrophages or Kupffer cells (KC), the liver sinusoidal endothelial cells (LSEC), the peri-sinusoidal hepatic stellate cells (HSC) formerly known as fat storing Ito cells, as well as liver lymphocytes particularly enriched in natural killer (NK) and natural killer T (NKT) cells. NPCs participate in normal physiologic liver functions and are also contributors to liver disease.

Liver injury is classified by duration into acute and chronic. Acute injury in its most extreme form results in acute liver failure (ALF), which is defined as an acute loss of hepatocyte mass (by necrosis or apoptosis) resulting in coagulopathy (elevated INR) and any degree of mental alteration (encephalopathy) in a patient without preexisting liver disease.^3^ Chronic liver injury is the result of long standing and ongoing liver damage and hepatitis from inflammation or intracellular stress responses (such as ER and mitochondrial stress). Common examples of chronic liver injury include steatohepatitis (alcoholic and non-alcoholic), autoimmune-hepatitis (AIH), primary biliary cholangitis, and chronic viral hepatitis (HBV, HCV). Acute and chronic liver injury results in hepatocyte death and turnover, which is commonly detected by an increase in the serum aminotransaminases (AST and ALT). If injury becomes chronic, the continuous cell death and turnover leads to chronic inflammation, activation of HSC into myofibroblasts and tissue repair responses, which result in fibrosis and ultimately cirrhosis and hepatocellular carcinoma. Controlling liver cell death both in acute injury to rescue the liver and in chronic injury to curb secondary inflammation and fibrosis is of paramount importance as a therapeutic strategy. When studying liver cell death, it is important to draw a distinction between acute and chronic liver disease models, since much of the controversy in the liver cell death field is because of these various contexts of injury under study. Different liver injury models differ in the type of signaling pathways activated, the role and the type of inflammatory cells involved, which ultimately dictate the cell death subroutine. For example, viral hepatitis results in cell death via death receptors (DR) and is executed mainly by adaptive cytotoxic T-cell activity resulting in liver cell apoptosis, whereas cell death from acetaminophen (APAP) hepatotoxicity, a hepatocyte intrinsic toxin, is largely necrotic and caspase and TNFR independent.^4–7^ This brings us to another point, and that is, although hepatocytes do express DR on their surface, not all liver cell death is DR-mediated and when comparing liver injury models one must take into account intrinsic death pathways activated by damage to the ER, mitochondria or nucleus, *versus* cytokine/DR ligand-activated DR signaling resulting in cell death.^8^

**Figure 1:**
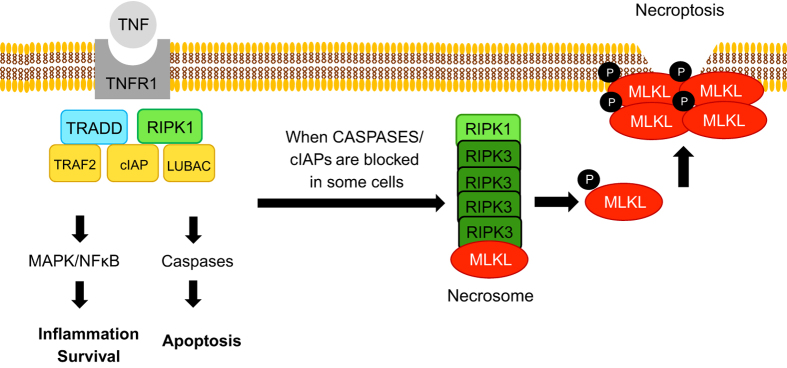
A simplified depiction of necroptosis induction by TNF. When TNF binds to its receptor (TNFR), complex 1 forms, which consists of the adaptor protein TRADD and ubiquitin ligases TRAF2 (also an adaptor), cIAPs, and LUBAC as well as the kinase, RIPK1. Ubiquitination of RIPK1 forms a platform to approximate key proteins leading to NF*κ*B and MAPK activation and transcription of pro-survival and proi-nflammatory genes. Complex I internalization leads to the formation of the cytosolic complex II (not shown), which ultimately results in Caspase-8 activation culminating in apoptosis. When caspases +/− cIAPs are inhibited in certain cells, RIPK1 then forms a complex with RIPK3, which oligomerizes and recruits the pseudokinase, MLKL. RIPK3 phosphorylates MLKL that activates the protein leading to its translocation to cell membrane where it forms tetramers to permeate the lipid bilayer. TNF, tumor necrosis factor; TRADD, tumor necrosis factor receptor type 1-associated death domain; TRAF2, TNF receptor-associated factor 2, cIAP, cellular inhibitors of apoptosis; LUBAC, linear ubiquitin chain assembly complex; RIPK1, receptor interacting protein kinase 1; NF*κ*B, nuclear factor *κ*B; MAPK, mitogen-activated protein kinase; RIPK3, receptor Interacting protein kinase 3; MLKL, mixed-lineage kinase domain-like.

Necroptosis, is a mode of regulated cell death initiated by DR and occurs when caspases (+/−cIAPs) are inhibited. In certain cells when these conditions are met, RIPK1 and RIPK3 interact via their RIP homology interaction motif (RHIM) domains to form a complex recruiting the psudokinase mixed-lineage kinase domain-like (MLKL). MLKL is activated and phosphorylated by RIPK3 and subsequently translocates to the cell membrane resulting in its rupture (Figure 1).^9^ In the past few years, much attention has been directed towards the role of necroptosis in human disease. This has also led to an increasing number of studies to explore this cell death mode in the liver using various animal models of acute and chronic liver injury; however, a wide range of conflicting conclusions have been generated (Tables 1,2,3). Below we attempt to address the known facts, the intriguing controversies and the pressing questions in the field of liver cell death with a focus on the necroptosis pathway.

## Do hepatocytes undergo necroptosis in models of liver injury?

### Hepatocytes do not express RIPK3 under basal conditions

As briefly described above, necroptosis is a form of programmed cell death often mediated by TNFR1 and other DR engagement with their ligands. It occurs through the RIPK1-RIPK3-mediated activation of MLKL in certain cell types when there is inhibition of caspases +/−cIAPs.^10^ Activated MLKL (p-MLKL) forms a tetramer that translocates to the cell membrane where it executes necroptosis by a mechanism, which is still under debate.^11–15^ MLKL driven rupture of cell membrane is the necessary end point of necroptosis and the only known activator of MLKL at present is RIPK3. Therefore, RIPK3 and MLKL are necessary for the induction of necroptotic cell death.^16^

Although hepatocytes clearly express MLKL, the presence of RIPK3 in liver cells has been controversial.^17^ RIPK3, the third member of the receptor interacting protein kinase family was first described in 1999.^18^ RIPK3 mRNA distribution was examined using Northern blotting and its presence in the liver was not conclusive in the original report.^18^ Kasof *et al.*^19^ further dissected the role of RIPK3 in apoptosis in 2000 and reexamined its tissue distribution and failed to detect RIPK3 mRNA under basal conditions in the liver. RIPK3 protein is weakly present in whole liver homogenate (mixed cell types) but this in fact may be because of its presence in the NPC compartment. Much of the controversy lies with its induction during injury, which has been based mainly on western blotting and immunohistochemistry (IHC) of RIPK3 protein using commercially available non-specific polyclonal antibodies on injured liver tissue. Interestingly, in most reports, the area of RIPK3 staining corresponds to the area of necrotic or damaged liver. Injured liver tissue is highly immunoreactive, we have observed positive staining with non-specific normal rabbit and mouse IgG.^17^ Furthermore, we have observed positive RIPK3 staining in livers of RIPK3−/− mice using commercially available polyclonal antibodies, confirming the lack of specificity of these polyclonal antibodies.^17^ To determine if hepatocytes express RIPK3, we isolated primary mouse hepatocytes (PMH) and immediately measured RIPK3 mRNA and performed western blotting on freshly isolated PMH using a monoclonal antibody to RIPK3 provided to us by Dr. Newton of Genentech. We could not detect RIPK3 in hepatocytes under basal conditions nor after cell culture with APAP or TNF at different time points. Nor could we detect an increase in RIPK3 in PMH isolated from mice treated *in vivo* with APAP for 3 h.^17^ Recently others have confirmed this data, reporting much lower RIPK3 expression in whole liver homogenate compared with other organs such as spleen and intestine and undetectable RIPK3 in isolated mouse hepatocytes.^20^ Whether hepatocytes express RIPK3 or not is of critical importance since, as far as we know now, cells that do not express RIPK3 do not undergo necroptosis.^21^ Of course, theoretically in some unique circumstances it is possible that MLKL can be activated in a RIPK3-independent fashion and this has been suggested to occur in immune-mediated liver disease, see ConA section for more details.^20,22^

### Blocking apoptosis in hepatocytes protects from cell death (with no switch to necroptosis).

Hepatocytes can die by necrosis and or apoptosis. In the liver, apoptosis is a common event in liver injury as hepatocytes robustly express DRs and many disease states have been shown to result in hepatocyte apoptosis, such as viral hepatitis (Councilman bodies), NASH (apoptotic bodies), alcoholic hepatitis, and autoimmune liver disease (adaptive immune-mediated).^23,24^ Direct DR activation under experimental conditions by TNF, FASL, TRAIL or DNA damage (ionizing radiation, cancer chemotherapeutic agents), or growth factor withdrawal have been shown to result in apoptotic cell death.^23^ Liver cells express DR (TNFR1, FAS, DR4/5), likely because of an evolutionary pressure to eradicate hepatotropic viruses. Indeed, during viral hepatitis T cells kill hepatocytes by DR-mediated apoptosis, which eliminates virally infected cells.^24^ Hepatocytes are type II cells and require the participation of the intrinsic mitochondrial apoptosis pathway for cell death completion.^25,26^ Only certain primary cells and cell lines such as L929 fibrosarcoma cells, Jurkat T cells, HT29 cells, MEF, keratinocytes express RIPK3 and have been shown to switch to an alternate form of cell death with DR signaling under conditions where caspases +/−cIAPs are inhibited.^21^ This has not been the case for all cells; for example, MCF-7, Hela cells and Hek293 cells do not undergo necroptosis because of a lack of RIPK3 expression.^21^ In fact, absence of RIPK3 in cells predicts whether they can undergo necroptosis.^27^ Under numerous apoptosis-inducing experimental conditions in hepatocytes we and others have observed a protection afforded by caspase inhibition without a switch to necroptosis.^28–35^ In addition, liver-specific ablation of caspase-8 has no effect on the normal liver and is in fact protective against Fas and TNF-mediated apoptosis.^32,36^ Therefore, the lack of a switch to a necroptotic form of cell death with caspase inhibition would argue against necroptosis occurring in liver cells. Necrosis of hepatocytes does occur in certain contexts but appears to be mediated by the mitochondrial permeability transition that can be considered a form of regulated necrosis. The MPT responds to ROS production and cyclophilin D controls pore opening.

## Acute liver injury models in which necroptosis has been studied

### The acetaminophen model

Acetaminophen (APAP) toxicity is a valuable model of liver cell death, hepatotoxicity and fulminant liver failure. The murine APAP model closely mimics human disease and is an excellent early translational model to study necrosis of hepatocytes. Once glucuronidation and sulfation pathways are saturated (in instances of overdose) APAP is metabolized to NAPQI via the Cyp450 system. NAPQI is highly electrophilic and covalently binds intracellular proteins and is thereby intrinsically toxic to hepatocytes. Inhibition or knockdown of many signaling molecules and MPT including components of the MAP Kinase pathway (MLK3, ASK1, JNK, Sab), GSK3b, PKCa, as well as cyclophilin D have been shown to protect against APAP.^37–42^ APAP-induced cell death has long been recognized to be necrotic and caspase and TNFR independent.^4–7^ It is a form of cell death which results in mitochondrial dysfunction and ROS generation, ATP depletion, MPT and necrotic oncosis. Given the involvement of signaling molecules, APAP hepatocyte death is a regulated form of cell death. However, as it pertains to necroptosis, it is important to point out that APAP cell death is independent of receptor signaling.^7,43^ Many investigators have evaluated the role of Necrostatin-1 (Nec-1) in APAP toxicity and have shown protection with the RIPK1 kinase inhibitor (Table 1).^17,44–48^ Yet, Nec-1 has off target effects and the true test of necroptosis is whether MLKL KO mice are protected against APAP. The effect of APAP on RIPK3 global knockout mice has been examined with varying conclusions. Two labs were unable to distinguish any protection against APAP toxicity at 24 h.^17,45^ However, Ramchandran *et al*.,^45^ observed protection against APAP (200–300 mg/kg) at 6 h but this protection did not extend to 24 h. We were unable to see any protection with APAP (300 mg/kg) using strain-matched controls at early or late times points.^17^ Deutsch *et al.*^46^ reported a robust protection against APAP (700 mg/kg) with a 75% survival advantage compared with C57BL/6j controls. In our hands 700 mg/kg of APAP results in 100% mortality in all WT and transgenic mice models (C57BL/6n).

The discrepancy in the reports may be in part because of experimental variations (such as APAP preparation methods) as well as strain matching. The Genentech generated RIPK3−/− mice are on an n sub-strain (personal communication with Genentech), whereas WT control mice used in the Ramachandran paper were j sub-strain and the C57BL/6 mice in the Deutsche paper were purchased from Jackson (likely j sub-strain unless otherwise requested). C57BL/6 substrains n and j have different susceptibility to APAP liver injury.^49^ Finally, given the controversy with the RIPK3 knockout mice and the fact that MLKL is indispensable for the execution of necroptosis, we examined the effect of APAP 300 mg/kg on MLKL−/− mice (compared with j sub-strain-matched controls) and found no attenuation of injury.^17^ This demonstrates that cell death in the APAP model is MLKL independent and thus most likely not necroptosis.^17^ Gunther *et al.*^20^ have independently obtained similar results, MLKL−/− mice were not protected against APAP compared with WT controls (j sub-strain).^20^ Although these results seem confirmatory, more rigorous studies using littermate controls and inducible liver cell-specific knockout are needed for conclusive results.^22^ Furthermore, we could find no change in RIPK3 expression in the short time course APAP toxicity (up to 24 h) *in vitro* or *in vivo*.

### Immune activation models: concanavalin A (ConA) and *α*-galactosylceramide (*α*-GalCer)

The plant lectin concanavalin A (ConA) is widely used to induce ‘immune-mediated’ acute liver injury in mice. It is often used as a model of hepatitis as the hepatocellular injury in ConA is primarily initiated by the direct activation of T cells, particularly CD4+ T cells and NKT cells and targeting of hepatocytes by the immune system in an antigen TCR-independent fashion.^50–54^ Subsequent activation of macrophages/KC and recruitment of leukocytes into the liver significantly contributes to the liver injury.^55,56^ The mode of cell death in ConA is largely necrotic, although some reports have observed DNA fragmentation and apoptotic bodies as early as 5 h after ConA injection, suggesting that apoptosis of hepatocytes occurs early in the injury phase followed by massive cell lysis and necrosis in the later hours.^57,58^ TNF is presumed to be the major cytokine activating hepatocyte DR as interference with TNF (treatment with anti-TNF antibodies) has been shown to be protective against ConA-induced hepatitis and hepatocellular death.^57–59^ TNF is directly capable of inducing hepatocyte apoptosis via TNF-receptor signaling inducing caspase-8 activation and involvement of the mitochondrial intrinsic death pathway, leading to mitochondrial cytochrome C release and caspase-3 activation. However, as TNF signaling also activates NF-*κ*B, which in turn switches on pro-survival gene expression such as cFLIP, BclXL among others, in order for TNF to mediate hepatocyte death, the deactivation or inhibition of the NF-*κ*B pathway is necessary. However, this does not seem to be the case with ConA as the lectin alone can result in hepatitis and cell death without the need for transcriptional or translational inhibition.^57^ In addition to TNF, other major cytokines such as IFN-*γ*, IL4, IL6 and other DR pathways (Fas, TRAIL) have also been implicated in ConA hepatotoxicity, although to a lesser extent than TNF.^60–65^ The ConA model is very complex. In addition to immune cells, LSECs have also been suggested to have a pivotal role in the injury by mediating a hypercoagulative state in the liver.^66^

In spite of the immune-mediated features of ConA toxicity, such as the requirement for TNF and IFN-*γ* signaling, liver cell death appears to be necrosis morphologically and not apoptosis. This is further supported by the fact that no significant activation of caspase activity has been observed with ConA and no protection from liver injury is afforded by caspase inhibition.^67,68^ Given the involvement of the TNF DR pathway and the late appearance of necrotic cell death in ConA model, multiple laboratories have investigated whether necroptosis and the RIPKs contribute to ConA-induced hepatitis (Table 2). Jouan-Lanhouet *et al.*^69^ examined the effect of necrostatin-1 (125 *µ*g) administration 15 min before on ConA (20 mg/kg, retro-orbital injection) induced hepatitis on liver damage using female C57BL6 mice and reported a decrease of about 50% in ALT and histologic injury *versus* PBS pre-treatment. Nec-1 is dissolved in DMSO, which is known to be hepatocyte protective, may have some confounding effects especially if not controlled for. They demonstrated that ConA-activated PARP-1 in the liver of WT mice and that the PARP-1 inhibitor, PJ-34, protected to the same extent as Nec-1. Nec-1 not only resulted in less hepatotoxicity as measured by ALT but also decreased PARP-1 activation, suggesting that PARP-1 may be downstream of RIPK1 kinase activation in ConA.^69^ Recently, the same group published a follow up paper, this time using 12 mg/kg of ConA and the same pre-treatment protocol with Nec-1 125 *µ*g, 15 min before ConA and they concluded that protection by RIPK1 inhibitor and PARP inhibitor was because of lower levels of IL-33.^70^ Zhou *et al.*,^71^ performed similar experiments on male C57BL/6 mice. They administered Nec-1 (1.8 mg/kg) or DMSO via tail vein 1 hour before ConA (20 mg/kg) and observed less AST and ALT as well as a survival benefit with Nec-1 pre-treatment (30% *versus* 7% mortality). Deutsch *et al.* while studying necroptosis in the ConA model observed that although RIPK3−/− mice were mildly protected as measured by ALT compared with WT C57BL/6, this did not translate to a survival advantage. However, interestingly, simultaneous treatment of WT mice with Nec-1 (1.6 mg/kg) and ConA (20 mg/kg) compared with ConA (20 mg/kg) alone exacerbated liver injury, more than doubling the ALT and resulting in 50% mortality from Nec-1 treatment.^46^

Deutsche *et al*. investigated the mode of cell death in ConA using a global knockout of RIPK3. Even though they interpret RIPK3 deletion as being protective, there was only a modest (<10%) decrease in transaminases.^46^ These minor differences in *in vivo* experiments using possibly non-strain-matched mice that are not littermates should be interpreted with caution. It is interesting that Deutsche and colleagues observed a robust aggravation of injury with Nec-1 and ConA co-treatment compared with ConA alone in contrast to findings by Zhou *et al.*,^71^ Jouan-Lanhouet *et al.*^69^ and Arshad *et al*.^70^ The protection reported with Nec-1 should also be further scrutinized given the possible off target effects of Nec-1.^72^ Interestingly, Weinlich *et al.*^73^ have also examined the effect of ConA on RIPK3−/− mice compared with WT littermates and noted that the knockout animals displayed the same level of liver damage as control littermates, strongly arguing against RIPK3-dependent necroptosis in ConA liver injury. Recently Gunther *et al.*^20^ have reported protection against ConA (25 mg/kg) in global MLKL−/− mice. Interestingly, whereas Nec-1 s (400 *µ*g i.p.) was also protective in their experiments, RIPK3 knockout was not. In addition, they noted an increase in both RIPK1 and MLKL protein after ConA, whereas RIPK3 protein levels, which are very weak in whole liver and absent in hepatocytes did not change or increase after ConA liver injury. It is unclear in these studies how MLKL is activated in the absence of RIPK3, but an unidentified kinase downstream of RIPK1 is suggested to contribute to this non-canonical necroptosis pathway which seems to be MLKL-dependent.^20,22^ Investigation of conditional and liver-specific knockout models is still warranted.

Another model of immune cell activation is *α*-galactosylceramide (*α*-GalCer), a specific activator for invariant NKT cells resulting in immune-mediated liver injury and induction of regulatory and inflammatory cytokines. In WT mice *α*-GalCer activation of NKT cells results in increased TNF, IFN-*γ*, IL4, and subsequent activation of innate immune cells in the liver, causing a mild to moderate liver injury (ALT ~300–1000 IU) at the peak time of 16–24 h. This immune-mediated model is quite similar to the ConA model, but relatively less severe and initiated by specific activation of NKT cells, which are uniquely abundant in the liver. Kang *et al.*^28^ have reported that both *α*-GalCer injury and ConA-induced liver damage is attenuated in global RIPK3−/− mice (ALT 200) accompanied by less TNF and IFN*γ* mRNA expression. To confirm that the RIPK3 deletion in NKT cells was attenuating the injury, they performed bone marrow (BM) transplants between RIPK3−/− and WT mice to generate chimeric animals. They showed that RIPK3 in BM cells was essential for liver injury to occur as ConA-induced liver injury was significantly attenuated in the WT animals that had RIPK3−/− BM. They provided evidence showing that the reduced liver injury in RIPK3−/− mice is not related to necroptosis of hepatocytes, and most likely because of an impairment of production of cytokines (TNF and IFN-*γ*) by RIPK3-deficient NKT cells in a necroptosis-independent fashion. Thus, these findings are very interesting but do not support the hypothesis of necroptosis occurring in hepatocytes as the RIPK3 deletion in NKT cells seems to be the limiting factor.^28^ We have also been interested in the *α*-GalCer model of liver injury and have examined the role of RIPK3 and MLKL deletion as well as RIPK1 knockdown via antisense oligonucleotide in NKT-mediated immune injury from *α*-GalCer. Contrary to the findings by Kang *et al.*, we found no difference in liver injury between global RIPK3−/−, MLKL−/− mice and strain-matched controls.^74^ However, we found that knockdown of RIPK1 markedly exacerbated *α*-GalCer-mediated liver injury and induced lethality as early as 6 h after treatment. Unexpectedly, despite the massive hepatocyte death and the effective RIPK1 knockdown (>95%) the exacerbated liver injury was not because of an impaired hepatic NF-*κ*B activation. As we observed no difference with RIPK3−/− or MLKL−/− mice, we concluded that necroptosis was not involved. We observed increased TUNEL staining and caspase-3 activation in the RIPK1 knockdown animals with severe liver injury and we suspected the mode of death was in fact apoptosis. On further investigation, we observed that the exacerbation of liver injury with RIPK1 knockdown was because of TNF-and caspase-dependent massive apoptosis of hepatocytes as neutralization TNF or inhibition of caspase by pan-caspase inhibitor zVAD.fmk conferred an almost complete protection.^74^ Interestingly, ZVAD protection was not accompanied by a switch to necroptosis.

## Chronic liver models

Alcoholic and non-alcoholic steatohepatitis (ASH and NASH) along with models of viral hepatitis are the most commonly studied chronic liver injury models. Studies of mechanisms of hepatocyte cell death in viral hepatitis have been somewhat restricted by the limited availability of *in vivo* animal models. However, studies of hepatitis C in cell culture have to a large extent concluded the mode of cell death as apoptotic.^75–77^ One study has reported an additive protection with *in vitro* utilization of Nec-1 to the pan-caspase inhibitor QVD, although this was examined on Huh7 cells, and not primary hepatocytes and there was no mechanistic exploration as to how Nec-1 was exerting its effects.^78^

Non-alcoholic fatty liver disease and steatohepatitis (NAFLD/NASH) are a leading cause of liver disease and obesity related mortality is the US with increasing prevalence worldwide.^79^ Chronic alcohol use also results in steatohepatitis and is an important cause of cirrhosis worldwide. Long standing steatohepatitis results in inflammation, cell death, fibrosis, leading to cirrhosis and hepatocellular carcinoma. Therefore, therapeutic strategies other than weight loss and abstinence from alcohol are sorely needed.^79^ So far, the mode of cell death in NASH is presumed to be apoptosis as interfering with apoptosis, caspase inhibition and liver-specific caspase-8 knockout greatly protect.^80^ However, studying animal models of steatohepatitis has proven to be challenging. Unlike the APAP model which very closely mimics human pathology, it is difficult to study alcoholic steatohepatitis (ASH) and NASH by replicating robust steatohepatitis in mice using alcohol or a high fat diet (HFD) alone. Mice do not develop the same degree of inflammation and fibrosis as humans which may be a function of the duration and intensity of alcohol or HFD feeding or normal physiologic metabolic differences.^81^ In humans, fatty liver and NASH are accompanied by visceral adiposity, insulin resistance, type II diabetes and metabolic syndrome, which has proven somewhat difficult to recapitulate in animal models. The same is true for alcohol, existing rodent models of alcoholic liver disease result in less liver injury from alcohol (lower inflammation, ALT and bilirubin) in mice and rats compared with the effect of alcohol on human liver.^82^ Nevertheless, using the available animal models the role of necroptosis has been investigated in ASH and NASH (Table 3).

Roychowdhury *et al.*^83^ examined the effects of RIPK3 in mice fed alcohol. They reported an increase in RIPK3 expression by IHC using a commercially available polyclonal antibody with the Lieber de Carli model of free access to alcohol as well as chronic binge feeding. The RIPK3 staining was more prominent around the central vein. In addition, the authors report RIPK3 induction in human liver biopsy samples of patients with alcoholic liver disease using IHC. In RIPK3−/− mice (back-crossed to j sub-strain) they reported a modest attenuation of liver injury compared with WT C57BL6/j sub-strain mice (ALT in WT of 40, AST 70 down to 20 and 40, respectively, for RIPK3−/−). They also reported less liver triglycerides, TNF and liver inflammatory cell numbers by IHC. Wang and colleagues used the Gao-binge model of alcohol feeding and reported an increased expression of RIPK3 using a (polyclonal RIPK3 antibody) with alcohol and protection from global RIPK3 deletion (ALT and steatosis) but no difference in hepatitis and neutrophil infiltration in the RIPK3−/− mice. They could not detect a transcriptional change (mRNA) in RIPK3 and attributed the increased protein levels of RIPK3 to impaired hepatic proteasome function because of less protein turnover resulting from alcohol feeding.^84,85^ Roychowdhury *et al.*^86^ have recently published a follow up paper to using a HFD model and comparing RIPK3−/− and WT controls. They find no protection against HFD-induced steatohepatitis with global RIPK3 knockout. In fact, they report worsening of injury compared with controls. The RIPK3−/− animals had basal insulin resistance, which significantly worsened on a HFD leading to steatosis, inflammation and fibrosis. It is interesting that RIPK3 knockout had diametrically opposite results in the alcohol model and HFD model experiments performed on the same mice by the same group. Even more intriguing is that in both instances the authors report increased RIPK3 in the liver by immunostaining.^83,86^ The chow-fed wild-type mice in the present study expressed no basal RIPK3 or p-MLKL, but after HFD feeding showed increased expression of both proteins by IHC. However, despite the high expression of these necroptosis proteins the mice had very little injury. Therefore it is highly unlikely for necroptosis to be activated in this model.^86^

Two groups have investigated the role of RIPK3 in liver injury, using the methionine choline-deficient model, which is used as a NASH model.^87,88^ Gautheron *et al*.^88^ demonstrate an increase in RIPK3 expression using IHC and western blotting (polyclonal antibody). They also reported that liver injury is worsened in the animals by caspase-8 liver-specific knockout. Interestingly, in the liver-specific caspase-8 KO increased hepatic RIPK3 expression was observed even in the control diet, which may be because of compensatory mechanisms that occur in embryonic knockout animal models, making them difficult to interpret. Alfonso *et al.*^87^ have used IHC on human livers with various diseases and have demonstrated an increase in RIPK3 staining in liver sections from humans with NASH, ASH and hepatitis B and C. One could interpret this one of two ways, either all of these human liver diseases result in RIPK3 upregulation and as the authors suggest an involvement of necroptosis, or the antibody is non-specifically binding injured liver tissue. The authors offer no explanation as to how caspases are inhibited in these disease states for necroptosis to ensue. In mice using the high fat choline-deficient and methionine deficient models, Alfonso and colleagues demonstrated an increase in RIPK3 and p-MLKL in the insoluble liver fraction and a protection from RIPK3 knockout. Curiously, although TNF (plus transcriptional or translation arrest) is known to induce apoptosis in hepatocytes,^8,89–91^ the authors were unable to prevent cell death from TNF/CHX by caspase inhibition. Both of these studies used the methionine and or choline-deficient models which are not ideal models of NASH because of a lack of insulin resistance and weight gain in the animals.^92,93^ Furthermore, the technical limitations and non-specific binding of polyclonal antibodies used in IHC of the liver, in our opinion, limits the conclusions one can reach from these descriptive studies.^17^

Regardless, assuming the RIPK3 induction in hepatocytes is indeed robust and relevant in chronic disease models, we are left with the dilemma of understanding how physiologic necroptosis occurs in the presence of caspases, as all models of necroptosis require the genetic deletion or inhibition of caspases +/− IAPs.^21,92^ One must also take into account that these models are global RIPK3 knockouts and it is not clear whether the observed protection from lack of RIPK3 is because of a hepatocyte intrinsic mechanisms or an effect on the immune system. In certain disease models, which exhibit hemorrhage or severe inflammation in the liver, the inflammatory cells and RBC infiltration to the liver could theoretically increase total RIPK3 levels because of the mixed cell population. Nevertheless, although we do not believe that RIPK3 is upregulated in in acute liver injury, we recognize that context is important and it is possible that in chronic models of liver injury RIPK3 is indeed induced in hepatocytes. Whether this has any relevance or correlation with necroptosis in the absence of caspase inhibition remains to be determined. Furthermore, one has to consider cell death-independent functions of RIPK3 expression in hepatocytes.

## Unclassified Drug-Induced Liver Injury

Wang *et al.*^15^ while testing a phospho-MLKL antibody as a specific necroptosis marker had reported positive staining in liver biopsy samples from patients with drug-induced liver injury (DILI). The authors however do not specify what kind of DILI samples they used and what drug or toxicant was the culprit. Drug-induced liver injury is a complicated clinical diagnosis with a wide range of presentations and a lack of objective diagnostic test, making it largely a diagnosis of exclusion.^94^ In the study by Wang *et al.*^15^ no clinical data or patient characteristics, pattern of injury (hepatocellular or cholestatic) or identification of suspected drugs was provided. It is extremely important to emphasize that although some drugs such as acetaminophen are direct hepatotoxins, most drug toxicities are idiosyncratic and HLA/adaptive immune-mediated.^95^ DILI presents in a diverse and heterogeneous fashion, some drugs target hepatocytes and cause a hepatocellular pattern of injury, whereas others result in cholestasis. Some drugs result in hepatocyte necrosis, some activate immune cells to target DR-mediated apoptotic cell death, whereas other drugs result in vanishing bile ducts and are mainly toxic to cholangiocytes. The authors report patchy p-MLKL staining of dead hepatocytes around the central vein area of the 14 reported ‘DILI patients’.^15^ This is an intriguing descriptive finding. However, there is not a mechanistic explanation. Furthermore, if this implicates necroptosis activation, one would have to assume that in these 14 patients, caspase activation was somehow inhibited or defective for the hepatocytes to not die of apoptosis as they are very prone to do and instead default to necroptosis. This scenario has yet to be shown in physiologic conditions.

## Remaining questions

### Does RIPK1 participate independent of the necrosome in hepatocyte death pathways?

Multiple groups have studied the role of Nec-1 in models of acute liver injury in particular APAP, and have observed protection. Given the off target effects of Nec-1, we examined the effect of RIPK1 knockdown using an IONIS antisense oligonucleotide which concentrates in the liver and knocks down RIPK1 with about 95% efficiency. As RIPK3−/− and MLKL−/− mice were not protected but knockdown of RIPK1 in these mice afforded partial protection against APAP necrosis, we believe RIPK1 may have a novel and independent function in hepatocyte death, which appears to be upstream of JNK. One of our most striking observations in the past few years was that knockdown of RIPK1 protected against hepatocyte death in the APAP toxicity model while worsening injury from immune-mediated liver injury caused by *α*-GalCer. These two models are vastly different. The former is a cell intrinsic toxin to hepatocytes, which mediates a cyclophilin D and MPT-dependent necrotic form of cell death and the latter a DR-mediated apoptosis of hepatocytes induced by activated-NKT cells. It should not be surprising that RIPK1, which is a key molecule in inflammatory pathways, functions both as a survival protein and a death-inducing protein in a context-dependent manner.^9,96,97^ Furthermore, RIPK1 has been suggested as a participant in ER stress-induced apoptosis,^98^ and it is likely that RIPK1 has additional functions beyond mediating apoptosis and necroptosis.^9^ We should note that although we did observe a protection against APAP with RIPK1 knockdown, others have observed no protection with embryonic RIPK1 liver-specific knockout.^99^ Although the antisense may have off target effects, there are also issues with embryonic knockout, namely the capacity of living organisms to compensate in unexpected ways for the loss of vital proteins and alterations in normal homeostasis. It remains to be seen if adult inducible liver-specific knockout of RIPK1 protects against any of the acute and chronic liver injury models and hepatocyte death.

### Does necroptosis occur in NPCs?

As discussed in the introduction, hepatocytes are not the only liver cell type. In fact, the liver has a rich and diverse microenvironment harboring multiple types of specialized cells. Hepatocytes function mainly to regulate metabolism, detoxify xenobiotics, synthesize critical circulating proteins, and generate and secrete bile acids. The NPCs, including the LSECs, KC, HSC, NK, NKT, are in constant cross talk with hepatocytes and each other. We have observed a robust expression of RIPK3 and MLKL in isolated LSECs, KCs and liver leukocytes.^17^ As NPC participate in normal liver functions and have all the required machinery for the execution of necropoptosis, it is intriguing to hypothesize that NPC necropoptosis may contribute to some forms of liver disease.

## Summary of Key Points

- RIPK3 is not expressed under basal conditions in hepatocytes.
- As MLKL is expressed in hepatocytes there may yet be a RIPK3-independent mechanism for activation of MLKL and necroptosis in these cells.
- RIPK3 may be induced in certain disease conditions of the liver, but if so, necroptosis-independent pathways must always be considered as well.
- Preventing apoptosis in hepatocytes after DR engagement does not result in a switch to necroptosis; rather it prevents cell death.
- Human liver disease conditions in which caspases are inhibited (a requirement for necroptosis) have yet to be reported.
- More conclusive littermate controlled studies are needed using cell-specific inducible knockouts of MLKL, and the RIPKs and valid antisera and reagents that are specific and suitable for immunoblotting to resolve controversies and uncertainties.

## Figures and Tables

**Table 1:** Publications concerning necroptosis in liver injury from acetaminophen toxicity

*Reference*	*Main findings*	*Comments*
*Acetaminophen (APAP) model*		
An *et al*.^44^	Nec-1 (1.65 mg/kg i.p.) 1hr before APAP protected mice RIPK1, RIPK3 induced with APAP	Likely polyclonal antibody (not disclosed) Do not detect RIPK3 at baseline Describe a higher molecular weight RIPK3 (incorrect MW for RIPK3) and hypothesize it is an oligomer and RIPK3 is induced Nec-1 s (more specific inhibitor) not used No effect on APAP metabolism (GSH unchanged between tx and con)
Ramachandran *et al*.^45^	RIPK3 morpholino protected against APAP 200 mg/kg at 6 h Nec-1 protected *in vitro* RIPK3 induction suggested with APAP RIPK3−/− protected at 6hr No protection at 24 h in RIPK3−/−	Polyclonal antibody Prosci Mismatched strain as controls and not littermate controls Used Morpholino to knockdown but was not detecting RIPK3 at baseline suggesting RIPK3 induction prevented with morpholino pre-treatment RIPK3 present at baseline inconsistently on blots Nec-1 s (more specific inhibitor) not used Only early time point protection No effect on APAP metabolism (GSH unchanged between tx and con)
Zhang *et al*.^47^	RIPK1 and RIPK3 induced with APAP Nec-1 0.125 mg/mouse (i.p) 30 mins before APAP 300 mg/kg protective Nec-1 pre-treatment survival benefit (No DMSO given to controls) Nec-1 post APAP protective at 4 h, 24 h	CD1 mice (all other papers used C57BL6 mice) Polyclonal antibody (Sigma, St Louis, MO, USA) DMSO not controlled for if Nec-1 dissolved in DMSO Nec-1 s (more specific inhibitor) not used GSH not different between Nec-1 pre-treatment plus APAP and APAP alone
Takemoto *et al.*^48^	IV nec-1 12.5 mg/kg or DMSO followed by APAP 800/kg after 15 mins (6 h time point) Nec-1 protects PMH *in vitro* against H2O2	Polyclonal antibody (Imgenex, Novus Biologics, Littleton, CO, USA) IHC staining necrotic areas Intravenous injection of Nec-1 Nec-1 s (more specific inhibitor) not used
Dara *et al*.^7^	RIPK3 not expressed in PMH RIPK3 abundant in NPC (KC and LSECs) RIPK3 not induced with APAP RIPK1 Knockdown (KD) with antisense protective against APAP RIPK1 induced or modified with APAP (band thicker) RIPK3−/− and MLKL−/− not protected Nec-1 (i.p.) protective *in vitro* and *in vivo* (pre-treatment)	Demonstrated polyclonal RIPK3 ab exhibits non-specific binding in injured tissue (IHC not specific) MLKL KO more definitive for necroptosis than RIPK3 Monoclonal anti-RIPK3 antibody Nec-1 s (more specific inhibitor) not used Used strain-matched controls, not littermates Antisense KD has potential pitfalls Not a metabolic effect, GSH unchanged between treatment (tx) and control (con)
Deutsch *et al.*^46^	RIPK3−/− protected at against APAP 500 mg/kg 12 h (only 20% less injury) RIPK3−/− had survival benefit after 700 mg/kg APAP Nec-1 (1.65 mg/kg) three doses protective before APAP 500 mg/kg	DMSO was not controlled for in Nec-1 experiments (known to be protective) Substrains unclear Polyclonal antibody (Abcam, Cambridge, UK) Confirmed results with Nec-1 s Neither GSH nor APAP adducts were measured to rule out effects on APAP metabolism Used littermates
Gunther *et al*.^20^	MLKL−/− mice not protected against APAP MLKL not induced after APAP RIPK3 not detected in hepatocytes Very low RIPK3 expression in liver compared with gut No RIPK3 mRNA increase seen with NASH or AIH in human liver samples (rtPCR) RIPK3 detected in KC	Did not look at RIPK3−/− and APAP; no effect of MLKL−/−
		
*Miscellaneous: unclassified* ‘*DILI*’		
Wang^15^	Phospho-MLKL antibody staining in human liver biopsy samples reported to have drug-induced liver injury (DILI)	Unclear clinical relevance: which drugs are implicated, and which pattern of injury (hepatitis or cholestasis) Descriptive Monoclonal antibody IHC stains positive in some liver cells on biopsy in a patchy and cytosolic distribution not cell membrane

**Table 2:** Publications concerning necroptosis in immune-mediated liver injury models

*Reference*	*Main findings*	*Comments*
*Immune activation models: concanavalin A (ConA) and* α*-galactosylceramide (*α*-GalCer)*		
Jouan-Lanouet^69^	Nec-1 (125 *µ*g I.V.) 15 mins before ConA 20 mg/kg protects at 10 h as well as PARP inhibitor pre-treatment	Used female mice (most studies have used male mice)
Zhou *et al.*^71^	Nec-1 (1.8 mg/kg I.P.) 1 h before ConA (20 mg/kg I.V.) improved mortality compared with ConA alone	
Weinlich *et al.*^73^	RIPK3−/− not protected from ConA (same as controls)	Used control littermates and saw no difference in ConA injury between WT and RIPK3−/−
Arshad *et al*.^70^	Nec-1 (125 *µ*g I.V.) given 15 mins before ConA (12 mg/kg I.V.) 10 h, 12 h, 24 h was protective, resulted in less injury, less inflammation and less IL-33	Immune cell activation in liver in Nec-1 pretreated animals was not different despite no control for DMSO except for neutrophils which were less with Nec-1 pre-treatment
Kang *et al.*^28^	Attenuated liver injury in global RIPK3−/− with *α*-GalCer	Global RIPK3−/− resulting in attenuated injury likely because of BM cells and not hepatocytes RIPK3−/− BM transplanted in Hepatocyte WT animals attenuated injury Not related to necroptosis but rather impairment of production of cytokines (TNF and IFN-*γ*) by RIPK3-deficient BM and NKT cells
Suda *et al.*^74^	RIPK1 knockdown worsens ConA (10 mg/kg I.V.) injury	Sub-strain-matched controls no difference between WT and RIPK3−/− (unpublished data)
Deutsch *et al.*^46^	Modest protection reported with RIPK3−/− in ConA (20 *µ*g/g I.V.) Nec-1 (1.65 ug/g, I.P.) pre-treatment daily×3 days, worsened ConA injury and diminished survival Nec-1 induced worsening of ConA injury was rescued by blocking apoptosis with a pan-caspase inhibitor	RIPK3−/− resulted in very modest protection against ConA (ALT 2800 *versus* 2000) RIPK3−/− slightly delayed death, but overall no mortality difference at 48 h RIPK3−/− had a better cytokine profile Confirmed results with Nec-1 s Single dosing of Nec-1 same effect as three doses before ConA Inconsistent findings between Nec-1 and RIPK3−/− may suggests this is a necroptosis-independent effect
Gunther *et al.*^20^	MLKL−/− protected against ConA (25 mg/kg, I.V.) Nec-1 s 400 *µ*g (I.P.) 30 min before ConA protected mice RIPK3−/− mice not protected against ConA RIPK1 and MLKL expression increased after ConA RIPK3 not detected in PMH and not upregulated after ConA	Mechanism of MLKL activation and translocation unclear (unidentified kinase suggested) RIPK1 upregulated but doesn’t activate MLKL Much higher dose of Nec-1 than others so difficult to compare
Suda *et al.*^74^	RIPK3−/−, MLKL−/−, RIPK1D138N (kinase dead) not protected against *α*-Gal same injury as strain-matched controls Nec-1 pre-treatment (1.65 mg/kg I.P.) did NOT protect against *α*-Gal RIPK1 knockdown markedly worsens injury (caspase-dependent and blocked by ZVAD, therefore apoptosis)	The cell death induced in RIPK1 KD mice by *α*-Gal was blocked by ZVAD (apoptosis) and there was no switch to necroptosis Kinase dead protein and necrostatin has no effect but knockdown of protein markedly induced apoptosis with *α*-Gal and worsens injury arguing for a role in the platform function Littermates not used (sub-strain-matched control) ASO may have affected NPC as well (confounding and off target effects)

**Table 3:** Necroptosis in chronic liver injury models

*Reference*	*Main findings*	*Comments*
*Chronic liver injury models: alcholic steatohepatitis (ASH) and non-alcoholic steatohepatitis (NASH)*		
Roychowdhury *et al.*^83^	RIPK3 expression increased in Lieber de Carli alcohol feeding model in mice and chronic plus binge alcohol feeding RIPK3−/− mice were protected from alcohol-induced liver injury, less TG, inflammatory cell numbers in IHC	Used a polyclonal antibody and mainly IHC (concerning for non-specific staining) Use female RIPK3−/− mice back-crossed to j sub-strain and compared with non-littermate j sub-strain controls Modest injury in this model makes finding differences difficult (ALT decreased from 40 in WT to 20 U/L in RIPK3−/− mice)
Gautheron *et al.*^88^	Used methionine- and choline-deficient diet (MCD) Mice with Caspase-8 liver-specific KO had worsening injury fromMCD diet which was ameliorated with global RIPK3−/−	MCD diet not true NASH Complicated multi knockout design with Caspase-8 being liver-specific and RIPK3 KO global complicates interpretation
Afonso *et al.*^87^	MCD diet Increase in RIPK3 staining in liver sections from humans with NASH, ASH and hepatitis B and C In mice treated with MCD diet, RIPK3 and p-MLKL induced and detected in the insoluble liver fraction Protection in RIPK3−/−	MCD diet not true NASH Either RIPK3 is induced in all liver diseases regardless of underlying mechanism of injury or non-specific staining due to use of polyclonal antibody Does not necessarily mean necroptosis is involved; may be due to inflammatory roles of RIPK3 Unable to prevent TNF-induced cell death in hepatocytes treated with TNF/CHX by caspase inhibition
Wang *et al.*^84^	Gao-Binge alcohol feeding model Global RIPK3 deletion offered protection in terms of ALT and steatosis but no difference in hepatitis and neutrophil infiltration in the RIPK3−/− mice	No transcriptional difference (mRNA of RIPK3 was not induced) but protein levels increased Polyclonal antibody used Suggest due to proteasomal turnover of RIPK3
Roychowdhury *et al.*^86^	High fat diet model (HFD) RIPK3−/− mice had worse injury compared with controls	In WT chow-fed mice no basal RIPK3 or p-MLKL but induction reported with IHC Complete opposite results to previous ASH model which could be due to pathophysiologic differences Male mice. Sub-strain matching likely although not reported (n or j)
